# Drinking Water Supply, Sanitation, and Hygiene Promotion Interventions in Two Slum Communities in Central Uganda

**DOI:** 10.1155/2018/3710120

**Published:** 2018-01-28

**Authors:** David Musoke, Rawlance Ndejjo, Abdullah Ali Halage, Simon Kasasa, John C. Ssempebwa, David O. Carpenter

**Affiliations:** ^1^Department of Disease Control and Environmental Health, School of Public Health, Makerere University College of Health Sciences, Kampala, Uganda; ^2^Department of Epidemiology and Biostatistics, School of Public Health, Makerere University College of Health Sciences, Kampala, Uganda; ^3^Institute for Health and the Environment, University at Albany, State University of New York, Albany, NY, USA

## Abstract

Poor water, sanitation, and hygiene (WASH) continue to contribute to the high prevalence of diarrhoeal diseases in low-income countries such as Uganda particularly in slums. We implemented a 3-year WASH project in two urban slums in Uganda with a focus on safe drinking water and improvement in sanitation. The project implemented community and school interventions in addition to capacity building initiatives. Community interventions included home improvement campaigns, clean-up exercises, water quality assessment, promotion of drinking safe water through household point-of-use chlorination, promotion of hand washing, and support towards solid waste management. In schools, the project supported health clubs and provided them with “talking compound” messages. The capacity building initiatives undertaken included training of youth and community health workers. Project evaluation revealed several improvements in WASH status of the slums including increase in piped water usage from 38% to 86%, reduction in use of unprotected water sources from 30% to 2%, reduction in indiscriminate disposal of solid waste from 18% to 2%, and increase in satisfaction with solid waste management services from 40% to 92%. Such proactive and sustainable community interventions have the potential to not only improve lives of slum inhabitants in developing countries but also create lasting impact.

## 1. Introduction

In many African countries including Uganda, there has been rapid growth of slum populations that are majorly situated in urban settings such as town and cities [1]. Slum dwellings are characterized by several problems that only vary in magnitude from one place to another including poor solid waste management; improper excreta and wastewater management; unequipped drainage especially of storm water; poor housing conditions; insufficient drinking water; unsafe food; poor vector and vermin control; and inadequate personal and general hygiene [2, 3]. The situation is aggravated by the fact that urban authorities lack the resources to satisfactorily provide required services and infrastructure [1]. As a result, slums have become breeding grounds for disease [2], making the search for solutions to improve health in such communities an utmost urgency.

In slum settlements of Kampala and Mukono located in central Uganda, the majority of community members have insufficient knowledge of the link between water, sanitation, hygiene, and health, evidenced by the epidemics of cholera and typhoid, and a high incidence of diarrheal diseases particularly in children under 5 years of age [4, 5]. Slum settlements in Kampala are among the areas that have experienced majority of the cholera outbreaks in Uganda [4], and the district registered a major outbreak of typhoid in 2015 [5]. Other health conditions common in slums which affect mostly children include malnutrition, malaria, and pneumonia. Available evidence shows that poor water, sanitation, and hygiene (WASH) plays an important role in the transmission of diarrheal diseases [6–8]. Moreover, cholera is almost always transmitted by consumption of contaminated drinking water and food [9]. The major factors leading to this contamination are low latrine and water coverage and poor domestic and personal hygiene practices [10]. As a result of poor planning, the location of pit latrines and other sanitary facilities is such that they end up contaminating drinking water sources [5]. Most latrine facilities used in slums are unimproved, privately owned, and are shared among several households [11]. Insecure tenure in these communities also contributes to low latrine coverage while the communal sharing of latrine facilities leads to their poor utilization and maintenance [11]. Evidence from systematic reviews and meta-analyses of WASH interventions in poor communities with unsafe water and inadequate sanitation has shown that interventions can reduce the risk of diarrheal diseases [6, 7] and water related diseases including ascariasis, dracunculiasis, hookworm infection, schistosomiasis, and trachoma [12, 13].

The envisaged plausible interventions to which this project was targeted were to seek solutions within the affected communities in order to improve the prevailing poor environment in slums as a deliberate effort to improve community health. This therefore necessitated the implementation of the project in two urban slum areas in Uganda with the aim of improving the health status of the inhabitants through conducting community proactive and sustainable interventions targeting two priority problem areas of access to safe drinking water and improved sanitation facilities. This paper describes the interventions that were undertaken by the project to improve the WASH status in two urban slums in Kampala and Mukono in central Uganda. The three-year project, which was carried out between 2010 and 2013, had three phases: preparatory, implementation, and evaluation as described below.

## 2. Preparatory Phase

### 2.1. Implementing Partners

The project was implemented through a partnership between Makerere University School of Public Health (MakSPH), Uganda; University at Albany, State University of New York, USA; and Tuskegee University, USA. Whereas MakSPH was responsible for the day to day running of activities, the US partners were involved in planning and management of the project. In addition, the US partners conducted visits to Uganda to support the Uganda team in implementation as well as for monitoring and evaluation purposes.

### 2.2. Project Sites

The project was implemented in two urban slum communities in central Uganda. These were Kikulu zone in Kawempe division, Kampala district, the country's capital city, and Kikooza zone in Mukono Municipality, approximately 20 kilometers from Kampala. Kampala and Mukono districts are among the most populated districts in Uganda and were in 2014 estimated to have populations of 1,516,210 and 599,817 persons, respectively [14]. Kikulu zone has an estimated area of 6 square kilometers with a population of 6,576, with three primary schools and one secondary school [15]. There are no government health units in the area with the nearest health unit located approximately 1.5 kilometers from the zone. The area is predominantly residential with several small businesses managed by residents. Kikooza zone is also predominantly residential with a few dwellers operating small scale trading businesses although agriculture is also conducted by some residents. The zone is located within one kilometer from Mukono town and is along the highway between Kampala and Jinja, a major town in the country. There are no government schools or health facilities in Kikooza; therefore residents have to travel for close to one kilometer to access the nearest facilities. However, private clinics and schools do exist in the area although services offered by such facilities are more costly compared to public ones.

### 2.3. Project Initiation

The project team held several meetings with local leaders, responsible health departments, and the general community to introduce the project to them. The project then established a field office in each study site to support implementation of activities. A community member was recruited from each area as a caretaker of the office and for community mobilization during project activities. The project initiation was important in ensuring that various stakeholders appreciated the project. In addition, the initiation prepared them to actively take part in the numerous project interventions.

### 2.4. Ethical Considerations

The project got ethical approval from Makerere University School of Public Health Higher Degrees, Research and Ethics Committee (101), as well as research clearance from the Uganda National Council for Science and Technology (HS 867). Several meetings were held with the community local leaders informing them about the project activities and all respondents who participated in surveys did so only after clearly understanding its purpose and giving written informed consent.

### 2.5. Baseline Survey

A baseline survey to assess the WASH status of the communities was conducted that involved both quantitative and qualitative methods. This survey involved 102 and 111 households in Kampala and Mukono, respectively. The baseline survey involved a household survey using a questionnaire administered among household heads or the next responsible adult found at home at the time of data collection, and an observational checklist used to observe parameters of interest at the household. The study questionnaire had questions on household water and sanitation, drinking water sources, bathroom and latrine, and rubbish disposal. In addition, the questionnaire assessed the household health seeking behavior and child morbidity for fever and diarrhea in the two weeks preceding the survey. The questionnaire also recorded social demographic characteristics of the household heads. On the other hand, the observational checklist was used to assess different WASH aspects including environmental sanitation, status of sanitary facilities, and state of water storage facilities. Household questionnaires were administered to respondents at selected households and, for each household, an observational checklist was filled. To select households, each zone was divided into five geographical clusters from which around 20 households were randomly sampled. In each cluster, the relative central point was the starting point from which data collectors then moved spirally outwards skipping one household. Only households with children below five years of age were eligible to participate in the survey. In the event that a household did not have a child under five years of age, the next household which met that criteria was selected. Additionally, 8 focus group discussions were conducted for community categories of adult male, adult female, male youth, and female youth, and 24 key informant interviews carried out with local leaders, teachers, health workers, and religious leaders. The survey also involved bacteriological analysis of drinking water from all households involved.

The baseline survey found that the major sources of water used by the community were piped water (38%) plus protected (30%) and unprotected springs (20%). In addition, the survey showed that although majority of households (86%) had latrines, the sanitary status found at most of them was poor. This included lack of hole covers (84%) and hand washing facilities (70%). The main method of solid waste disposal was dumping in open pits then burning (55%) while others buried it (11%), disposed of it indiscriminately (18%), or used skips (7%). Only 40% of the households were satisfied with the solid waste management services in their community. In addition, 48% of households lacked dustbins while more than half (55%) did not have external drying racks for their utensils. Among households, 40% disposed their waste water in drainage channels and 33% in their backyard while only 2% used soak pits. Furthermore, although the majority of households (95%) claimed to treat their drinking water, boiling (94%) being the most commonly used method, only 39% samples had water with no* Escherichia coli*. Most households (54%) also kept their drinking water on floor level.

Results from the baseline survey (including the qualitative component) also revealed gaps in WASH including poor water quality of the sources used by the community; poor sanitation status including the sharing of latrines by some households and their poor maintenance; poor solid waste management; and poor knowledge, attitudes, and practices regarding WASH among community members. This therefore provided the basis for implementation of the project in these areas. The information obtained from the baseline survey was crucial for designing the WASH interventions for the slum communities as part of the project and were used as reference for later evaluation. The baseline survey findings were shared with the respective communities, their leaders, and other stakeholders through meetings, routine visits, reports, and training sessions.

## 3. Implementation Phase

### 3.1. Project Interventions

The project carried out several WASH interventions within the project sites working hand in hand with various stakeholders like the community, health practitioners, local leaders, community health workers (CHWs), and teachers among others. The project activities were categorized into community interventions, school interventions, and capacity building initiatives as elaborated below.

### 3.2. Community Interventions

#### 3.2.1. Home Improvement Campaign

The project worked with community mobilizers and CHWs to implement home improvement campaigns in the area. This involved carrying out house to house visits to ascertain the WASH status of households. Based on findings of the inspections, the team would sensitize household members on the ideal requirements in a home. This activity was useful to households as they were told the WASH shortfalls in their homesteads and advised of the appropriate measures to improve the situation. The major concerns established at households during the inspections were maintenance of latrines, personal hygiene, solid waste management, and water quality and drainage.

#### 3.2.2. Clean-Up Exercises

The project team supported the communities in routine clean-up exercises in their area to improve sanitation through sensitization, mobilization of community members, and provision of necessary equipment such as rakes, brooms, slashers, and personal protective wear notably gloves and gumboots. The clean-up exercises involved collection of wastes from the community, sweeping the area, digging and desilting drainage channels, and slashing overgrown vegetation among others. These activities were carried out by the community members themselves with the project team only providing support including necessary logistics. This improved the aesthetic appearance of the areas and reduced waste in the community. Such waste would otherwise be a source of vector breeding in the community such as flies and rats.

#### 3.2.3. Improvement of Water Sources and Water Quality Assessment

The project team carried out routine sanitary inspection of water sources to identify hazards in the water supply system that had the potential to contaminate them. They then, together with the community members, worked hand in hand to eliminate such hazards and reduce chances of water contamination. This activity included regular improvement of the drainage of the sources as well as educating the users on the proper use of facilities. The project also carried out regular water sampling and analysis from randomly selected communal and household sources and supported interventions to improve water quality. The findings from water quality analysis were regularly shared with community members. Individuals responsible for sources that were found to be contaminated were advised on respective interventions including treating drinking water, for example, by boiling. This ensured that the community accessed and utilized water from safer sources.

#### 3.2.4. Promotion of Household Chlorination

The project partnered with Uganda Health Marketing Group (UHMG), a nongovernmental organization, to promote use of chlorine tablets (Aquasafe™) as an option of treating drinking water at households. This involved conducting training sessions on the use of Aquasafe and subsequently promoting its use in the community. This method, which was unknown by many community members, was greatly appreciated particularly by households that had difficulty in boiling water for drinking. Indeed, the use of Aquasafe greatly increased in the communities during the project which further promoted drinking safe water.

#### 3.2.5. Promotion of Hand Washing

**Figure 1:**
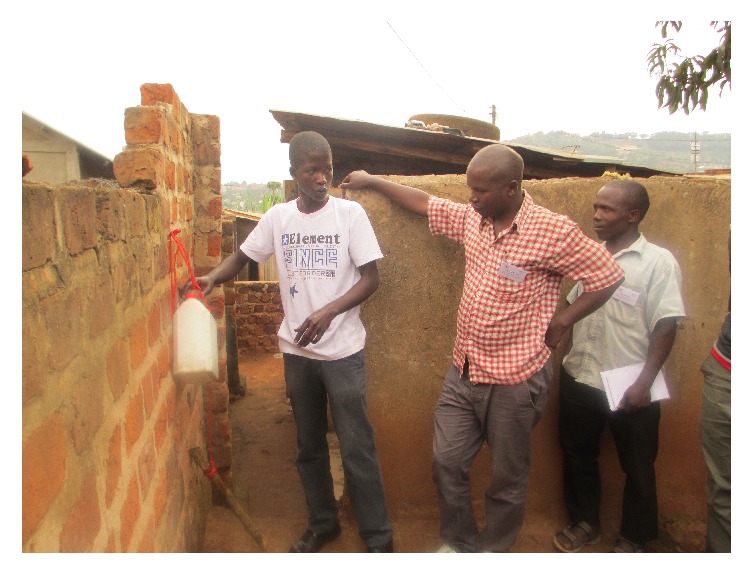
A youth teaches colleagues how to make a* tippy tap*.

The project promoted hand washing with soap at critical times particularly before eating food and after visiting the latrine. Special training sessions on hand washing, specifically, the use of* tippy tap* technology were conducted in the community (Figure 1). The* tippy tap* is a simple device for hand washing with running water made of locally available materials. It consists of a container that holds water, which is tipped by a foot-operated stick and rope tied through a small hole in the container cap for water to flow during hand washing. Following the training sessions, over 200 households in the study areas constructed these hand washing facilities at their latrines with support of the project. The introduction of the* tippy tap* technology greatly improved the hand washing practices of the general community including children who enjoyed using the facility after latrine use.

#### 3.2.6. Support to Waste Management

At the start of the project, the baseline survey had established challenges in solid waste management in the slums. The project therefore promoted resource reuse and recovery from household waste. These included waste separation at point of generation, composting for biodegradable wastes, and reuse for plastics. During dissemination of baseline findings and subsequent training, the community was educated on the preferred options of waste management, including recycling. The project then supported a youth organization (Youth Centre Support Network) to reduce waste in the areas by collection of plastics. This support included the provision of protective equipment like gloves and gumboots as well as training of youth involved regarding ideal ways of waste management. In addition, the community was sensitized about the dangers of poorly disposed plastics. The collection of plastics greatly improved the management of solid wastes as fewer plastics were available in the general waste stream after the intervention. The plastics were later sold to a recycling plant in Kampala, from which they earned some income.

#### 3.2.7. Stool Examination among Children

In the first year of the project, continuous stool sample collection and examination from children under 5 years with diarrhea was conducted. Based on the results, children were referred to nearest health facilities for treatment. However, this activity was not sustainable as the community's expectations of providing medicine to the sick children could not be met by the project. Nevertheless, families whose children were examined were appreciative of this initiative by the project. In addition, the project increased awareness on the need to have children with diarrhea examined by health workers as soon as they were ill.

#### 3.2.8. Advisory Roles in WASH

The project provided advisory roles in WASH to the project communities. This included routine visits to the communities where meetings were held to discuss WASH challenges that were being faced. These meetings were attended by the project team, local leaders, CHWs, and other health authorities in the area. This helped support the community and provided information necessary to promote WASH in the area. The expertise of the project team in WASH was particularly important in supporting improvement in the area.

### 3.3. School Interventions

#### 3.3.1. Support to Health Clubs

The project supported health clubs in two primary schools in the project communities. These schools were Kikulu primary school (Kampala) and Lweza primary school (Mukono). The project conducted several WASH activities among the health club members of these schools including training, health education, demonstrations, and drawing competitions. The training of these pupils not only benefitted them but also colleagues in addition to their family members. The health club members were WASH ambassadors in the schools who played a crucial role of promoting proper sanitation and hygiene practices among pupils. The patrons of health clubs, who were school teachers, were also involved in the activities. At the end of the project, 200 health club pupils were rewarded for their participation in the activities with school bags having health messages. The messages on the bags such as “keep your environment clean” also promoted WASH in the schools.

### 3.4. “Talking Compound” Messages in Schools

**Figure 2:**
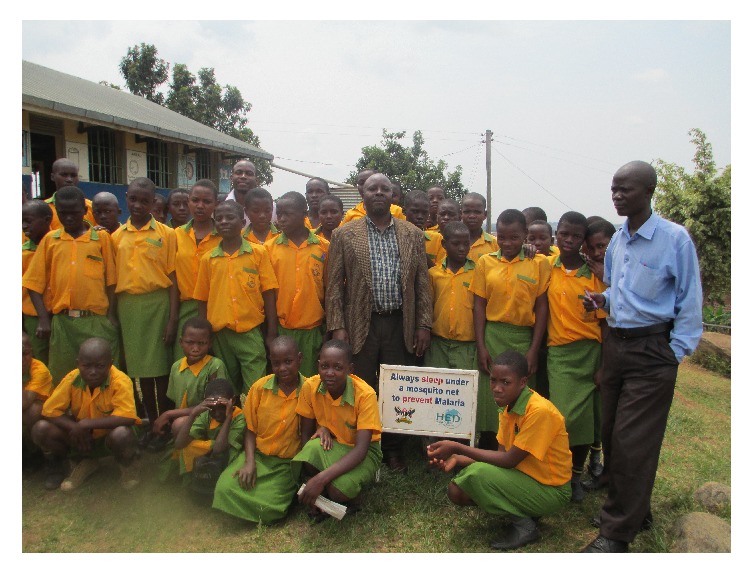
Members of a health club from one of the schools together with their teachers and project staff at one of the “talking compound” messages.

To promote appropriate WASH practices in the two schools, the project supported the development of “talking compound” messages which were displayed in appropriate places in the compound (Figure 2). The messages were as follows:* “Wash hands after visiting the toilet”; “Sleep under a mosquito net”; “Wash your hands before eating food”; “Remove stagnant water from your compound”; and “Keep the environment clean.”* These messages helped promote sanitation and hygiene among the entire school population and were very popular among the pupils and teachers. These messages also acted as reminders for the pupils to always observe proper sanitation and hygiene practices.

### 3.5. Capacity Building Initiatives

#### 3.5.1. Capacity Building of Youth in WASH

**Figure 3:**
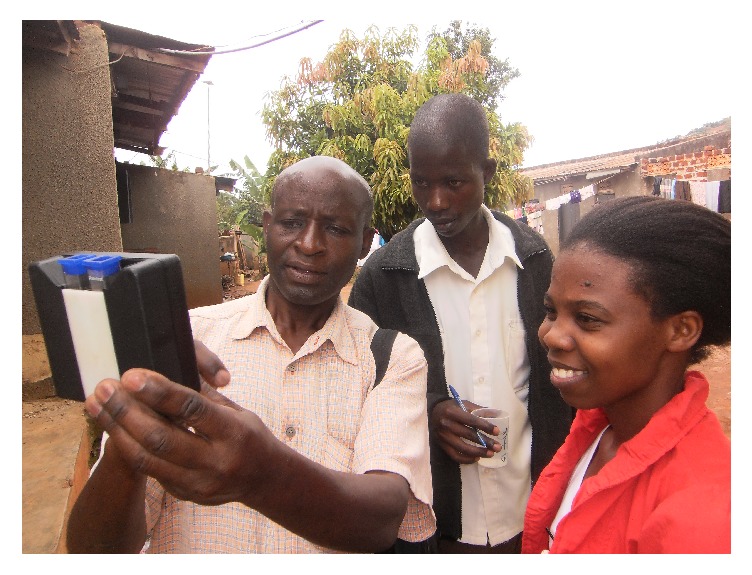
A youth (centre) being taught how to measure residual chlorine by Makerere University faculty.

Several youths in the project areas were trained in WASH techniques including inspection of households and water sources, use of water testing equipment, and research (Figure 3). They then worked hand in hand with project staff to implement interventions and in mobilizing the community for various activities. This helped build the capacity of the youth in the project sites as a sustainability strategy. Following the training and mentorship offered to the youth, they were able to carry out WASH improvement activities in the community on their own including household inspections.

#### 3.5.2. Training Community Members in WASH

**Figure 4:**
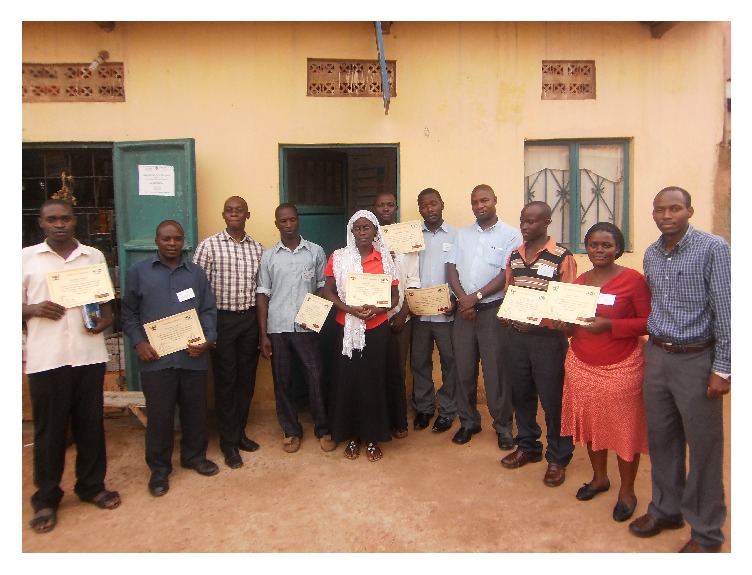
Trainees with their certificates after completing the WASH training.

The project trained 41 community members in a two-day short course in WASH. Those trained were community health workers, local leaders, and individuals involved in community work in their area. The trainees were responsible for promoting WASH in their respective communities through health education, house to house visits, clean-up exercises, and improving solid waste management among other responsibilities. These activities were to be carried out not only during but also after the project. The training sessions were facilitated by faculty of Makerere University School of Public Health and conducted in the community. The trainees received certificates on completion of the training (Figure 4) and t-shirts. The t-shirts were particularly important for identity during their health promotion work in the community.

#### 3.5.3. Exchange Visits

The project organized visits between members of the two study sites (project mobilizers and community health workers) to learn from each other and share experiences. These were crucial for each group to appreciate the challenges faced by the other slum areas as well as understand how the other groups promoted WASH in their communities. These exchange visits also provided an opportunity for learning how the other community had implemented project interventions. This fostered mutual learning between the project mobilizers and CHWs and further built their capacity in WASH. It was evident that although both communities were slums, there was a lot to learn from each other including approaches used to address WASH challenges. For example, one of the sites was turning solid waste into briquettes for use as cooking fuel which was taught to their counterparts.

#### 3.5.4. Capacity Building of Makerere University Faculty

Several staff members of the Department of Disease Control and Environmental Health at Makerere University School of Public Health were involved in various project activities as a form of capacity building. These staff included lecturers, research fellows, and laboratory and administrative assistants. The activities staff were involved in included health education, community and school training, research, and provision of advisory roles in WASH to the community. This also gave the faculty an opportunity of closely working with a community as well as appreciating the challenges faced by urban slums in the country. The experiences gained by the faculty were also useful during their teaching of students at the university as well as research. One faculty member was also sponsored as part of the project to study an M.S. in Environmental Health at the University of Albany, USA.

#### 3.5.5. Strengthening Laboratory Capacity at Makerere University

The project procured two microscopes and three pieces of bacteriological water testing equipment for Makerere University to support laboratory work during the implementation of project activities including water quality testing and stool examination. This equipment strengthened laboratory capacity and continued to be used by students of Environmental Health Sciences at Makerere University to conduct practical sessions. Faculty also used the equipment during their research activities even beyond the project.

#### 3.5.6. Research and Learning

**Figure 5:**
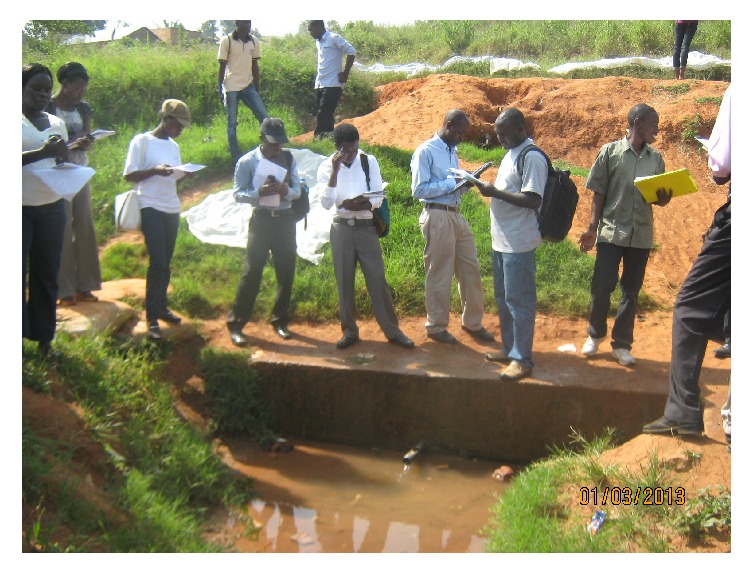
Bachelor of Environmental Health Sciences students inspecting a water source.

The project supported research activities carried out in the communities including a research that assessed the community knowledge, attitudes, and practices on solid waste management. This study provided information that was useful in advising the community on how to improve on waste management in their area. In addition to community dissemination, results of the study were also shared with the wider scientific audience through a peer-reviewed publication [3]. The project also supported 2 postgraduate students from United Kingdom (UK) who carried out their academic research within the study area. One student from Nottingham Trent University, UK, studied the role of CHWs in community development while the other from Trinity College, Dublin, Ireland, assessed the hand washing practices of the community. In addition, the project supported Environmental Health students at Makerere University to gain field exposure by their involvement in project activities (Figure 5). The activities they were mainly involved in included household visits, sanitary inspection of water sources, and promotion of hand washing using* tippy taps*. This field exposure was very important to the students to add to the theoretical knowledge that they had been taught in class. The project areas were also used as field sites for other students who were interested in learning about project activities including those studying a certificate course in WASH at Makerere University School of Public Health.

#### 3.5.7. Project Sustainability

Several proactive sustainable interventions were implemented during the project. These included the capacity building of youths and CHWs through training and exposure in WASH. The project also utilized already existing CHWs which was a means of further building their capacity and ensuring continuity in their work as these are charged with health promotion at the community level and are the first point of contact with the health system in such areas. The project also ensured that implementation of interventions was led by the community itself which led to ownership of the project and contributed to its continuity. Additionally, the project worked together with local stakeholders such as local and religious leaders, nongovernmental organizations, and the community in all project phases which further promoted ownership and support for project interventions. All these interventions were geared towards ensuring that project interventions were sustained beyond the project lifespan.

## 4. Evaluation Phase

### 4.1. Project Evaluation

After the project implementation period, a final evaluation survey was carried out among 300 households (150 from each site) in the community. The survey was cross-sectional in design and involved both quantitative and qualitative data collection methods. The same questionnaire and observational checklist that had been used during the baseline survey was utilized for the end term evaluation. From these households, the household heads or their spouses answered the questionnaire and observations were made of the sanitary and environmental hygiene of the household. To determine households to participate in the survey, each study site was divided into four (4) geographical clusters from which an average of 38 households was sampled from each. In each cluster, the relative central point was the starting point and moved spirally outwards skipping one household. All households in the area were eligible to participate in the survey. Also, drinking water samples were collected from households involved in the survey. In addition, 24 key informant interviews were also carried out with local leaders, school teachers, and health workers, and 8 focus group discussions were conducted with community members separate for adult women, adult men, female youths, and male youths.

The evaluation revealed several improvements in the WASH status of the slum communities. Indeed, piped water usage improved from 38% to 86% with a reduction in the use of unprotected sources from 30% to 2%. Treatment of drinking water improved from 95% to 99% with more households (96%) boiling their water than 94% who did so at the baseline. Additionally, latrine coverage improved from 86% to 99% and the status of the latrines also improved. Indiscriminate disposal of solid waste reduced from 18% to 2% and satisfaction with solid waste management services increased from 40% to 92%. The number of households with no dustbin reduced from 48% to 30%, though those without utensil drying rack increased from 55% to 70%. In addition, presence of soak pits for management of waste water at households increased from 2% to 10% and fewer households disposed waste water in their backyard, from 33% to 27%.

The evaluation survey including the qualitative component revealed that there were significant improvements in the WASH status of these communities after the implementation of several multifaceted interventions. These improvements were in WASH practices, for example, hand washing, boiling of drinking water, management of solid waste, cleanliness of drainage channels, water sources management, environmental hygiene, general cleanliness of the area, and cleanliness of sanitary facilities. There was also a reported reduction in diarrheal diseases among the study communities qualitatively. Findings from the project evaluation were widely shared with community members through meetings, routine visits, and reports. Findings were also shared with the wider audience through conferences, exhibitions, and seminars.

## 5. Challenges Faced in Implementation of Project Activities

### 5.1. Migration in and out of the Community

One of the major challenges the project faced was the frequent migration of people in and out of the areas. Being residents of slums, some of the population were not permanent residents and hence moved to other areas whenever there was a need, for example, in search for jobs. This negatively affected the project as some of the members trained left the community. This challenge was mainly addressed by selecting members for the major project training who had lived in the area for a long time and were not expected to leave the community.

### 5.2. Undesirable Community Behavior

In certain circumstances, the behaviors of some members of the community were undesirable regarding implementation of project activities. For example, even with extensive mobilization, some members of the community would not turn up for health education sessions. This was because, being residents of slums, some of the population felt less concerned with community activities as it was not their area of birth/home village. This problem was also more pronounced in Kikulu slum located within the capital city compared to the Kikooza slum in Mukono district. However, the many people who attended these sessions were expected to pass on the messages to other members of the community. A few community members also stole the* tippy taps* from some latrines in the area. This was either to use them at their own latrines or use the jerrycans for other purposes. This forced some community members to lock the jerrycan inside the latrine as opposed to having it installed outside the facility. Although the community was extensively sensitized on the importance of these hand washing facilities, some members still continued with this negative practice.

### 5.3. Unfavorable Weather

During some times of the year, bad weather notably heavy rains could not enable carrying out certain project activities such as house to house visits and other field activities. As soon as the weather improved, the project team together with the other stakeholders including the community was able to implement the pending activities planned for the respective periods.

### 5.4. Poor WASH Infrastructure

The project areas had poor WASH infrastructure including few and unprotected water sources, dilapidating housing, poor state of latrines, lack of provisions for solid waste management, for example, skips, and insufficient health facilities. These infrastructural challenges negatively affected implementation of certain project interventions; for example, some wells were dilapidated that mere community cleaning and drainage improvement would not improve quality of the water. In addition, even after community sensitization to improve solid waste management practices, there were not enough skips for waste collection from the community yet the scope of the project did not include provision of such WASH infrastructure/facilities.

## 6. Discussion

This project demonstrated how the partnership between US universities and one in Uganda helped improve the lives of residents in urban slums through implementation of proactive and sustainable WASH interventions with full community involvement. Indeed, the project mainly provided support to community members who then actively participated in many activities such as clean-up campaigns, improvement of water sources, and installation of handwashing facilities at their households. Community participation in WASH projects increases ownership of the interventions and generates a positive attitude towards practices [16]. In addition, community projects with elements of social action tend to amplify impact by providing benefits at both community and individual household levels and support local governance to oversee interventions [17–19], which are important in ensuring the sustainability of project interventions. The project also trained CHWs, local leaders, and youth in the communities so as to take lead in implementing project activities which was crucial in the success observed and can be replicated in other programs. In addition, training of CHWs ensured that individuals already involved in health promotion were better equipped to improve the health and wellbeing of their respective communities, which together with capacity building of youth and local leaders was crucial in ensuring that WASH interventions would continue to be implemented after the project duration. This stresses the importance of building sustainability components in WASH interventions early enough and throughout project implementation so that there is continuity even after projects end. In fact, sustainability of interventions is considered the ultimate test that all development projects should strive to achieve [20, 21].

The health clubs in the two primary schools greatly benefitted from project activities in the schools. The “talking compound” messages at the schools were also key in reminding the pupils and promoting ideal practices for the improvement of their hygiene and health. It was therefore a beneficial strategy to not only work with the general community but also promote sanitation and hygiene in schools. The pupils were always enthusiastic and eager to learn probably since they were members of health clubs with interest in health issues. It was noted that the sessions conducted in the schools as part of the project not only benefitted the trained individuals but other pupils in the school as well as family members. Indeed, previous research has demonstrated that school WASH interventions targeting children can also have an impact on their parents and the wider community [22–24]. Future projects implementing WASH interventions could utilize schools as an avenue for having a significant impact in the community.

The project had a significant impact at institutional level besides the community. At Makerere University, the laboratory capacity was greatly strengthened by the acquisition of water testing equipment and microscopes which were nonexistent before the project. This greatly supported the Bachelors program in Environmental Health Science as well research by staff at Makerere University School of Public Health. Students involved in the project were also eager to learn about project activities in the field as well as take part in planned activities which benefitted them through obtaining field exposure at the sites and carrying out internship in the community. The communities benefitted from the various project activities which improved their knowledge, attitudes, and practices in WASH. This certainly improved their lives and hence contributed to reduction in the occurrence of diarrhea and related diseases as has been demonstrated in previous studies [6, 25, 26].

## 7. Limitations

Although the project focused on capacity building (training, sensitization, and health education), the communities also had challenges in WASH infrastructure including few and unprotected water sources, dilapidating housing, poor state of human excreta disposal facilities, and insufficient health facilities. These communities would therefore benefit from future interventions targeting improvement of such infrastructure. Even if this was beyond the scope of the project, it was appreciated as a need that could be addressed to further improve the lives of the inhabitants of these (and other) slums in Uganda. In addition, the project was implemented in only two urban slums. However, it is worth noting that Kampala alone as a district has over 10 slum communities. This shows that there is a lot of potential to replicate project interventions in other slums. This project was clearly a pilot with its success being critical for use in improving WASH in other slum areas in Uganda and sub-Saharan Africa.

## 8. Conclusion

The project improved the WASH status of communities in two urban slums in Uganda through implementation of proactive and sustainable interventions both in the community and schools. Urban slums can benefit from WASH interventions when communities are fully involved in all stages of implementation with a focus on capacity building. The achievements of the project can inform the design and implementation of future interventions to improve WASH in urban slums in developing countries.

## Abbreviations

CHW: Community health worker
MakSPH: Makerere University School of Public Health
UHMG: Uganda Health Marketing Group
WASH: Water, sanitation, and hygiene.
